# Recent Developments in Yeast Aging

**DOI:** 10.1371/journal.pgen.0030084

**Published:** 2007-05-25

**Authors:** Matt Kaeberlein, Christopher R Burtner, Brian K Kennedy

**Affiliations:** Massachusetts General Hospital, United States of America

## Abstract

In the last decade, research into the molecular determinants of aging has progressed rapidly and much of this progress can be attributed to studies in invertebrate eukaryotic model organisms. Of these, single-celled yeast is the least complicated and most amenable to genetic and molecular manipulations. Supporting the use of this organism for aging research, increasing evidence has accumulated that a subset of pathways influencing longevity in yeast are conserved in other eukaryotes, including mammals. Here we briefly outline aging in yeast and describe recent findings that continue to keep this “simple” eukaryote at the forefront of aging research.

## Introduction

**Figure 1:**
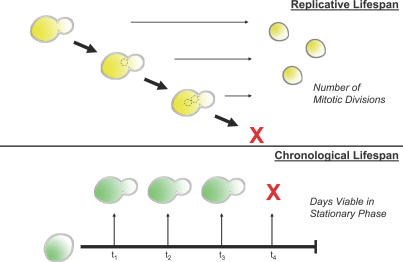
Schematic for Yeast Replicative and Chronological Aging (A) RLS in yeast is measured by the number of mitotic divisions that can arise from a single mother cell. Replicative viability is calculated as the mean number of daughters produced from mothers of a particular strain background before senescence. (B) CLS is measured by the length of time cells in a stationary culture can remain viable. Viability is calculated by the fraction of the culture able to reenter the cell cycle after an extended state of quiescence.

The budding yeast Saccharomyces cerevisiae is a widely used model of cellular and organismal aging [1–4]. The first studies of yeast aging were published over 50 years ago, in which yeast cells were shown to have a finite replicative capacity [5]. Replicative life span was thus defined as the number of daughter cells produced by a mother cell before senescence. A second model of aging has more recently been developed in yeast, termed chronological aging. In contrast to replicative life span (RLS), chronological life span (CLS) is defined as the length of time a yeast cell can survive in a nondividing state [6]. These two models for aging in yeast (Figure 1) provide a unique opportunity to compare and contrast the aging processes of both proliferating and nonproliferating cells in a simple single-celled organism [3].

An interesting parallel has emerged from studies in both yeast aging models linking environmental nutrients to longevity. In the lab, yeast cells are typically grown in media containing high levels of glucose (2%) and abundant amino acids. Independent studies have determined that reducing either the glucose or amino acid concentrations of the media (or both) can increase replicative and chronological life span [7–12]. These different nutrient restriction paradigms have all been referred to as calorie restriction. Calorie restriction is known to increase life span in a variety of organisms other than yeast, including worms, flies, and rodents [13,14]. Given that there is some debate about whether the life-span benefits of these interventions are a direct result of reduced caloric input [15–17], we have chosen to use term dietary restriction (DR) hereafter. There is much interest in determining whether the mechanism(s) by which DR increases longevity in yeast are evolutionarily conserved. A major focus of yeast aging research recently has been directed at understanding the mechanisms that underlie life span extension by DR in yeast.

## Dietary Restriction and Sir2: Still Looking for Consensus

Much of the popular interest in yeast aging over the past several years has developed from studies of the silent information regulator 2 (Sir2) family of protein deacetylases (sirtuins). A role for sirtuins in longevity determination was first suggested from work showing that deletion of *SIR2* shortens replicative life span [18], while overexpression increases replicative life span [19]. Sir2 orthologs have since been reported to play a similar role in determining the longevity of both worms and flies [20, 21].

In yeast, both overexpression of *SIR2* and deletion of *FOB1* repress homologous recombination at rDNA repeats. Recombination of rDNA results in the accumulation of extrachromosomal rDNA circles, which can lead to replicative senescence [22]. While it was initially proposed that DR increases RLS in yeast by activating the Sir2 enzyme [11], this model has been challenged by a series of recent studies demonstrating that DR can increase RLS by a SIR2-independent mechanism [23–25]. Although DR fails to increase RLS in a *sir2Δ* mutant, DR robustly increases the RLS of *sir2Δ fob1Δ* double mutant cells, demonstrating that Sir2 is not required for life span extension by DR [24]. It remains controversial whether the Sir2 ortholog Hst2 could mediate RLS extension by DR in yeast under specific DR conditions when Sir2 is absent [26,27]; however, recent findings indicate that DR can increase RLS through a mechanism that is independent of all yeast sirtuins [28]. Arguments regarding the relevance of Sir2 in DR have been covered in greater detail in recent reviews and commentaries, and we refer the interested reader to these sources [29–31].

In the chronological aging paradigm Sir2 does not promote longevity and appears to play an antagonistic role in the response to DR [8]. Unlike RLS, deletion of *SIR2* does not shorten CLS under normal growth conditions [8]. When cells are subjected to DR, deletion of Sir2 significantly increases CLS [8]. One mechanism that has been proposed for this antilongevity function of Sir2 involves regulating the expression of alcohol dehydrogenase, which is important for metabolism of ethanol late in stationary phase [8]. Whether additional functions of Sir2 are involved as well, such as its role in partitioning of oxidatively damaged proteins between mother and daughter cells [32], remains to be determined.

## Apoptosis and Oxidative Stress: Cause or Effect?

An interesting question that has emerged from recent studies is the potential relationship between an apoptosis-like pathway and cellular senescence in yeast. Morphological and molecular features resembling apoptosis in metazoan cells were initially reported in a S. cerevisiae strain with a point mutation in the *CDC48* gene [33]. Since this original characterization, apoptotic phenotypes in yeast have been reported to occur under a variety of conditions, including overexpression of pro-apoptotic mammalian BAX [34], by transfer of a stationary culture to a glucose medium lacking additional nutrients to support growth [35], and by treatment with low concentrations of H_2_O_2_ [36].

Of particular interest to the field of aging is the effect of oxidative stress on aging cells. Reactive oxygen species accumulate during the diauxic shift and stationary phase, as cells switch from fermentation to oxidative phosphorylation [37]. Reactive oxygen species are also potent stimulators of the mitochondrial cell death pathway, causing loss of mitochondrial membrane potential and export of cytochrome C. Interestingly, either deletion in the yeast ortholog of the apoptosis-inducing factor *AIF1* [38] or overexpression of anti-apoptotic mammalian BCL2 [39] rescues the apoptotic phenotype induced by ROS.

With regard to these recent advances, the link between yeast apoptosis and aging remains correlative, but highly intriguing. Both replicatively and chronologically aged cells show markers consistent with apoptotic death [37]. At present, however, it remains unclear whether there exists a causal relationship between apoptosis and senescence in yeast. It has been reported that deletion of the yeast caspase *YCA1* results in an increase in chronological longevity, but only after the culture falls below 10% viability [40]. This suggests that apoptosis exerts an effect on longevity only after the majority of cells have already undergone senescence. Thus, for most cells in the population, activation of the apoptosis-like pathway may be a response to the damage leading to senescence. The observation that apoptotic markers are present in both replicative and chronologically senescent cells may be an indication that the ultimate cause of senescence is similar in both dividing and nondividing yeast cells. This would be consistent with the finding that chronologically aged cells have a reduced RLS [41] and that some interventions (e.g., DR or reduced target-of-rapamycin (TOR) signaling) increase both RLS and CLS (see below).

It has also been suggested that apoptosis-like events in chronologically aged cells provide an opportunity for a few individual cells within the population to resume cell division. This argument is based on observations that, at a low frequency, vegetative growth will occasionally be observed late in stationary-phase cultures [42]. It has recently been speculated that this “gasping” effect is an altruistic phenomenon, whereby the majority of cells in an aging population die through a process resembling apoptosis in order to facilitate the outgrowth of a few remaining viable cells [42]. This hypothesis, although controversial, provides an interesting link between replicative and chronological longevity and also suggests a potential mechanism for how an apoptosis-like pathway might evolve in a single-celled eukaryote.

## Genome-Wide Screens Elaborate the Importance of Nutrient-Responsive Kinases

An important advance in the field of yeast aging over the last two years has been the development and application of genomic methods for assaying longevity [12,25]. Previously, studies of aging in yeast had been limited to a relatively small number of genes and relied on biased approaches: testing of candidate genes based on prior knowledge or assumptions and screening for secondary phenotypes, such as stress resistance [18,43] or age-associated changes in gene expression that may correlate with longevity [44].

Two reports from genome-wide studies of yeast aging, carried out using a collection of ~4,900 isogenic single-gene deletion strains [45], have identified the nutrient-responsive TOR signaling pathway as an important mediator of both replicative and chronological life span [12,25]. Mutations that decrease TOR activity were found to increase the longevity of both dividing and nondividing yeast cells [12,25]. Interestingly, decreased TOR activity also increases life span in both worms and flies [46–48], suggesting an evolutionarily conserved role for TOR as a conduit linking nutrient status to longevity.

In yeast, TOR acts in concert with other nutrient-responsive kinases, Sch9 and protein kinase A (PKA), to coordinate the cellular response to altered glucose and nitrogen levels [49,50]. Prior studies had implicated roles for both Sch9 and PKA in yeast aging. Similar to inhibition of TOR, deletion of Sch9 increases both replicative and chronological life span [25,43,51]. Likewise, a temperature-sensitive allele of yeast adenylate cyclase *(cyr1–1)* that decreases PKA activity increases both replicative and chronological life span [9,43]. Suprisingly, deletion of small G proteins that activate the PKA pathway, Ras1 and Ras2, results in opposite effects on the chronological and replicative life spans; deletion of *RAS1* increases RLS while slightly decreasing CLS, while deletion of *RAS2* decreases RLS, but dramatically extends CLS [9,51,52]. Thus, multiple studies have independently uncovered an important role for these nutrient-responsive signaling pathways in determining yeast longevity.

**Figure 2:**
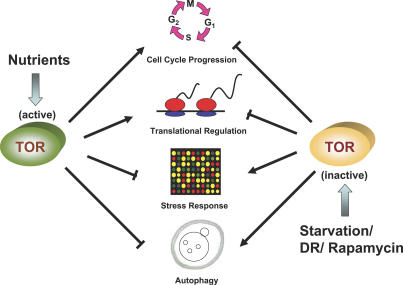
TOR Kinase Mediates Important Cellular Responses Implicated in Extended Longevity During periods of nutrient availability, TOR kinase is activated, leading to G1 progression, translation initiation, increased ribosome biogenesis, and a suppression of autophagy and the stress response. When TOR is inactivated, either by DR or by the TOR inhibitor rapamycin, a cellular response is initiated that turns down protein translation and cell growth, and increases protein turnover and genes involved in the stress response. The conserved cellular regime that is the result of inactivated TOR kinase increases both replicative and chronological life span in yeast.

How might decreased activity of nutrient-responsive kinases lead to increased life span? TOR, Sch9, and PKA play overlapping regulatory roles in several cellular processes that could be of relevance for longevity (Figure 2). In the remainder of this review, we consider which downstream functions are most likely to determine longevity in yeast and, potentially, other organisms.

### 

#### Stress response.

One function of TOR, Sch9, and PKA is to repress a general stress response by regulating localization of the transcription factors Msn2 and Msn4 [53–56]. Under conditions of high nutrient availability Msn2/4 are retained in the cytoplasm, where they are unable to activate transcription of starvation-induced stress proteins [53]. Under starvation conditions, or upon treatment with the TOR-inhibitor rapamycin, Msn2/4 relocalize to the nucleus, resulting in enhanced resistance to oxidative and temperature stress. Extension of CLS by the *RAS2* deletion appears to be due in part to Msn2/4 activation [9]; however, the chronological life span extension imparted by deletion of Sch9 is independent of Msn2/4; instead, it partly involves activation of *RIM15* [43], which mediates entry into stationary phase and activation of stress-responsive genes under those conditions [57].

Although it has not been demonstrated that activation of Msn2/4 is sufficient to either increase replicative or chronological life span, there is indirect evidence supporting the idea that Msn2/4 are involved in chronological life span extension from TOR inhibition. For instance, overexpression of the Msn2/4 target genes *SOD1* and *SOD2* is sufficient to increase chronological life span [9], suggesting that decreased TOR activity results in increased chronological life span, at least partially, through upregulation of superoxide dismutase activity. Thus, one mechanism by which decreased nutrient availability might slow chronological aging is through an upregulation of stress resistance via activation of Msn2/4 and other pathways.

Interestingly, replicative life span extension from *SCH9* deletion, mutations reducing PKA activity, or DR is not dependent on Msn2/4 [11,58]. Thus, given the current available data, the Msn2/4-mediated stress response appears to play an important role in nutrient-mediated chronological, but perhaps not replicative, life-span extension in yeast.

#### Retrograde response.

The retrograde response has been defined as a mitochondrion-to-nucleus signaling pathway that is activated in response to mitochondrial dysfunction [59,60]. This process is mediated by the transcription factors Rtg1 and Rtg3, which coordinate expression of enzymes involved in anapleurotic production of α-ketoglutarate. The retrograde response has been previously implicated in yeast replicative longevity, with the observation that deletion of mitochondrial DNA (rho^0^) can increase life span in a retrograde-dependent manner [61]. The relevance of this finding has been difficult to determine, however, because deletion of mitochondrial DNA increases replicative life span in only one out of the six yeast strains in which it has been studied [23,52,61].

In addition to mitochondrial dysfunction, however, retrograde gene expression is also regulated by TOR activity, and treatment of cells with rapamycin induces Rtg1/3-target genes [62,63]. Thus, it is reasonable to speculate that one mechanism by which TOR inhibition could influence replicative longevity is by altering retrograde gene expression. It will be of interest to determine whether Rtg1 and Rtg3 are required for replicative or chronological life span extension from TOR inhibition. Interestingly, deletion of the retrograde target gene *IDH2,* coding for isocitrate dehydrogenase, also increases yeast replicative life span [25] and two different isocitrate dehydrogenase enzymes are reported to similarly affect longevity in C. elegans [64]. Thus, there is evidence that altering the expression of TOR-regulated retrograde target genes can influence longevity.

#### Autophagy.

Yet another important function of TOR proteins is to repress autophagy [65,66]. Autophagy is a starvation response in which cellular macromolecules are recycled through vesicular transport and degradation in lysosomal or vacuolar compartments [67]. Autophagy has been implicated in age-associated disease, and autophagy decreases as a function of age in rodents [68–71] More recently, it was shown that increased autophagy is required for full life-span extension in C. elegans in a long-lived *daf-2* mutant [72].

Although direct experimental data is lacking, autophagy could be an important mediator of yeast longevity, particularly chronological life span. It is known that yeast cells upregulate autophagy during entry into stationary phase, presumably as an adaptive response to starvation [66]. Consistent with this, several mutants defective for autophagy are short-lived in the chronological aging assay [12]. Treatment of yeast cells with rapamycin or growth under nitrogen starvation induces autophagy [66] and also increases chronological life span [12]. Enhanced autophagy could have several beneficial properties in aging post-mitotic cells, including degradation of oxidatively damaged proteins, inhibition of protein aggregation, and recycling of damaged mitochondria. It will therefore be of interest to determine whether increased autophagy is important for life-span extension from DR or TOR inhibition in either or both of the yeast aging models.

#### Changes in carbon metabolism.

Yeast cells have evolved to undergo a variety of metabolic changes in response to fluctuating nutrient levels in the environment, many of which are coordinated by TOR, Sch9, and PKA. In particular, yeast respond robustly to decreasing glucose levels by shifting their metabolic state from one that favors fermentation to one that favors respiration. It has been proposed that this shift in carbon metabolism may account for the increase in RLS observed in response to DR [73]. The mechanism postulated by this model was that enhanced respiratory activity would activate Sir2, thus increasing life span. Contrary to this hypothesis, DR increases the RLS of respiratory-deficient cells [23]. This is true, even in cells completely lacking mitochondrial DNA. Similar to the case with *SIR2* and DR, there continues to be disagreement about the requirement of respiration for life-span extension by DR, and it has been recently reported that deletion of *LAT1,* which encodes a component of the mitochondrial pyruvate dehydrogenase complex, also is required for life span extension by DR [74].

There is additional evidence that changes associated with respiratory metabolism can influence both RLS and CLS. For example, overexpression of the glucose-repressible gene *HAP4* is sufficient to increase both RLS [73] and CLS [4], even when glucose levels are high. Hap4 is a regulatory subunit required for optimal transcriptional activation by the Hap2/3/5 complex, which induces respiratory genes in response to the available carbon source. Putative Hap2/3/5 binding domains have also been identified in the *TSA2* (thiol-specific antioxidant) promoter, which responds to increased oxidative and nitrosative stress [75]. In that report, overexpression of *HAP4* was demonstrated to induce *TSA2* expression. Thus, in addition to inducing respiration, *HAP4* is important for promoting cellular stress resistance. It remains to be determined whether the effects of *HAP4* overexpression on replicative and chronological longevity are related to its effects on respiratory metabolism or a different function.

#### Decreased ribosome biogenesis and translation.

One of the primary functions of TOR, Sch9, and PKA is to modulate protein translation in response to environmental cues [49,76,77]. In yeast, one mechanism by which these kinases regulate translation is by promoting transcription of ribosomal proteins (RPs) and rRNA processing factors. Under conditions of glucose or nitrogen starvation, or upon inhibition of TOR with rapamycin, RP transcription is dramatically reduced and translation in general is impaired [49,63,76,77].

A link between TOR, RPs, and longevity was suggested from the initial results of a genome-wide screen for replicatively long-lived mutants. Replicative life span was determined for 564 single-gene deletion strains randomly chosen from the yeast ORF deletion collection, resulting in the identification of 13 long-lived mutants [25]. In addition to *TOR1,* the deleted genes from these 13 long-lived strains included two TOR-regulated RP genes, *RPL31A* and *RPL6B* **.** We have since determined that several other RP and rRNA processing factor deletion mutants are also long-lived (our unpublished data), and Chiocetti et al. [78] recently reported that *RPS6B* and *RPL10* similarly regulate replicative longevity. These findings suggest the possibility that one mechanism by which decreased TOR activity can increase replicative life span is by decreasing ribosome function and translation. In this regard, it is noteworthy that mutations in S6 kinase, a downstream target of TOR involved in ribosome maturation, have been reported to increase life span in flies [47], and several recent reports have implicated mRNA translation as a critical determinant of longevity in worms [79–81].

### From Yeast to Mammals

It remains an open question how much of the aging process will be conserved from yeast into higher organisms. Clearly, some aspects of aging in yeast are specific to yeast. Others, however, appear to be highly conserved. Life span extension from Sir2-overexpression, TOR-inhibition, Sch9/Akt or DR, for example, has been observed in yeast, worms, and flies. It is likely that several additional conserved longevity factors will be identified from ongoing genome-wide screens in yeast and worms [12,25,52,64,82–85] and studies in mammalian models. If a given gene functions similarly to regulate longevity in yeast, worms, and mice, there is a good chance this function will be conserved in humans. In this way, yeast may serve as a foundation for identifying potential targets for intervening in human longevity and age-associated disease.

The observation that TOR, Sch9/Akt, and PKA could be regulating longevity differently in replicative and chronologically aging yeast cells is noteworthy, given that DR appears to retard a variety of age-associated diseases in tissues of higher animals [14]. The beneficial effects of reduced nutrient signaling may be dependent on the proliferative state of the tissue in question in mammals. Mice subjected to DR are resistant to carcinogenesis and display reduced age-associated pathologies in brain, liver, heart, muscle, and other tissues. How is it that DR has such a broad spectrum of beneficial effects in complex organisms? Based on the studies in yeast described above, we speculate that a few key nutrient-responsive proteins (such as TOR) may serve as evolutionarily conserved gatekeepers to synthesize inputs from the environment into appropriate tissue-specific outputs. For example, in neuronal cells, enhanced degradation of aggregated proteins through increased autophagy might be of particular relevance, whereas in fibroblasts increased resistance to stress or appropriate modulation of ribosome function could be most important. Future studies of the differential responses of different cell types to DR and TOR inhibition will be important for testing this idea.

A growing body of evidence clearly suggests that aging is determined, at least in part, by ancestral evolutionary origins. Due to this conservation, yeast remains a powerful tool for dissecting the genetic and biochemical factors that modulate longevity. As large-scale screens for long-lived yeast deletion mutants draw closer to completion, new and unexpected pathways are being uncovered, bringing a global picture of cellular aging into sharper focus. The knowledge gained from the molecular biology of aging in yeast yields a foundation on which to approach the puzzle of multicellular aging in tissues and in higher organisms.

## Abbreviations

CLS: chronological life span
DR: dietary restriction
PKA: protein kinase A
RLS: replicative life span
RP: ribosomal protein
Sir2: silent information regulator 2
TOR: target of rapamycin
